# A Comprehensive Study of Bacterial Etiological Agents in Sterile Body Fluids and Antimicrobial Susceptibility Patterns Among Hospitalized Patients at an Academic Medical Center in India

**DOI:** 10.7759/cureus.71862

**Published:** 2024-10-19

**Authors:** Rounak Patel, Satyajeet Pawar, Satish Patil

**Affiliations:** 1 Department of Microbiology, Krishna Institute of Medical Sciences, Krishna Vishwa Vidyapeeth (Deemed to be University), Karad, IND

**Keywords:** antimicrobial susceptibility pattern, bacteriological profile, gram negative cocci, gram positive bacilli, multidrug resistant organisms, sterile body fluids

## Abstract

Background

Sterile body fluids are devoid of any microbial presence, including commensal bacteria. However, bacterial invasion of these fluids can result in life-threatening infections, often leading to significant morbidity and mortality. Timely detection and precise identification of pathogens, along with antimicrobial susceptibility testing, are critical for optimizing therapeutic interventions and improving patient outcomes.

Objective

To study the prevalence of bacterial infections in various body fluids in hospitalized patients and to determine the antimicrobial susceptibility pattern and the phenotypic detection of extended-spectrum beta-lactamase (ESBL), metallo-beta-lactamase (MBL) and AmpC beta-lactamase producers within bacterial isolates.

Materials and methods

Sterile body fluid samples, excluding blood and urine, were collected and cultured at the Department of Microbiology, Krishna Institute of Medical Sciences, Western Maharashtra, India, from November 2022 to 2023. The microorganisms isolated from these fluids were identified using standard biochemical tests. Antibiotic sensitivity was assessed through the disc diffusion assay (zone of inhibition) and phenotypic identification of beta-lactamase enzymes was performed using the combined disc diffusion method.

Results

During the study period, 180 sterile fluid specimens were collected representing 48 cerebrospinal fluid (CSF), 53 pleural fluid, 23 peritoneal fluid, and other sterile body fluid samples. Out of these, (n=32, 17.77%) samples were culture-positive. Gram-negative bacteria were oftentimes isolated at 84% (27/32), while gram-positive were 16% (5/32). *Escherichia coli* was frequently isolated and (n=9, 28.12%) exhibited maximum sensitivity to gentamicin and fosfomycin (n=7, 77.78%) and maximum resistance to cefoperazone-sulbactam (n=8, 88.88%). *Pseudomonas aeruginosa* was isolated as the second most common organism and showed maximum susceptibility to fosfomycin (n=5, 83.34%) and maximum resistance to gentamicin, cefotaxime, cefoxitin, etc. (n=5, 83.34%). Among gram-positive isolates, coagulase-positive *Staphylococcus *was high in prevalence rate and (n=3, 9.37%) presented 100% sensitivity to vancomycin and maximum sensitivity to tetracycline (n=2, 66.67%) and 100% resistance to ciprofloxacin, cefoxitin, erythromycin, and other antibiotics. Among gram-negative isolates, MBL producers were 48.15%, ESBL producers were 40.74%, and 18.51% were AmpC beta-lactamase producers with a multidrug-resistant (MDR) occurrence rate of 93.75%.

Conclusion

Infections affecting sterile body fluids are critical due to their high mortality and morbidity rates. Timely identification of the causative organisms and their antibiotic susceptibility is essential. The prompt initiation of appropriate antibiotic therapy can decrease the duration of hospitalization and mitigate the emergence of drug resistance. The presence of MDR organisms in sterile body fluids constitutes considerable challenges in the management of critically ill patients.

## Introduction

As per the World Health Organization (WHO), the term sterility is defined as the absence of viable microorganisms; thus, sterile body fluids are biological fluids devoid of any microorganisms, including bacteria, which are neither present as commensal nor as part of the normal microbial flora [1,2]. Body fluids, including cerebrospinal fluid (CSF), pleural fluid, pericardial, bile, peritoneal or ascitic fluid, and synovial fluid, are generally classified as sterile body fluids as they lack viable microorganisms under normal circumstances [3]. However, bacterial multiplication in these sterile body fluids causes infections that may become life-threatening and result in significant morbidity and mortality [4]. Early detection and identification of organisms from infected body fluids, along with antimicrobial sensitivity testing, is important for the proper management of the patients. The prevalence of bacterial growth is usually low in various body fluids due to a smaller number of pathogens and previous management with empirical antibiotics in these patients. Even a single colony isolated from these specimens is considered a pathogenic microorganism [5,6].

Furthermore, many pathogens isolated from body fluids may be drug-resistant. The emergence of multidrug-resistant (MDR), extensively drug-resistant (XDR), and pan-drug-resistant (PDR) pathogens, as well as methicillin-resistant *Staphylococcus aureus* (MRSA) and extended-spectrum beta-lactamase (ESBL) producers, complicates clinical management in patients [5]. The prevalence of body fluid infections may increase in resource-constrained settings as a result of reliance on empirical treatment practices [7].

The existing literature on bacterial infections of body fluids and their antimicrobial susceptibility patterns in our region is limited. This study aims to identify bacterial etiology and their antimicrobial susceptibility in hospitalized patients at an academic medical center, thereby enhancing early diagnosis, ensuring appropriate treatment, and preventing antibiotic resistance.

## Materials and methods

Study location, type, and duration

The study was undertaken at the Department of Microbiology, Krishna Institute of Medical Sciences, Karad. A cross-sectional hospital-based analysis was carried out from November 2022 to November 2023.

Sample size calculation and sampling method

The sample size for this study was determined using the formula n = 4pq/I^2^, where p presents the prevalence of bacterial growth, q is calculated as 100 - p, and I denotes precision. As per the study conducted by Sharma et al., prevalence was 30% [8].

n = 4pq/I^2^

= 4 × 30 × 70/72

= 171

Thus, a minimum of 171 fluid specimens was determined as the sample size for this study. The sampling method employed was consecutive sampling. To strengthen the study, 180 sterile body fluid specimens were studied.

Inclusion and exclusion criteria

All the specimens of sterile body fluids, except for urine and blood, were included from patients of all age groups and genders. Patients with a history of antibiotic use two weeks prior to specimen collection, as well as those with delays in fluid transport exceeding two hours, were excluded.

Ethical clearance

The study was approved by the Ethical Committee of Krishna Institute of Medical Sciences, Karad, under protocol number 069/2021-2022, reference number KIMSDU/IEC/04/2022, and approval date 09/05/2022. 

Specimen collection and processing

After receiving approval from the Ethics Committee of the institute, specimens of sterile body fluids, including CSF, pleural fluid, pericardial fluid, bile, peritoneal (ascitic) fluid, and synovial fluid were collected after obtaining patient consent. All specimens were collected in sterile containers and transported to the microbiology laboratory within two hours.

In the laboratory, all specimens were investigated using standard microbiological methods. Initially, a smear preparation was performed for gram staining using the direct specimen, followed by inoculation on enriched media such as blood agar and heated blood agar as well as differential media like MacConkey agar (Hi-Media, Mumbai, India) using the four-quadrant streak method. The inoculated plates were incubated at 37°C for 24 hours and then observed for bacterial growth the following day. All bacterial growth was examined for colony characteristics, including gram staining, motility, and biochemical tests for species identification. Samples were considered sterile only after 48 hours of incubation [9].

Antimicrobial susceptibility test

The antibiotic susceptibility testing was conducted for all isolates using the Kirby-Bauer disk diffusion technique in accordance with Clinical and Laboratory Standards Institute (CLSI 2023) guidelines [10]. For gram-positive bacteria, the antimicrobials used included ampicillin (10 μg), gentamicin (10 μg), ciprofloxacin (5 μg), tetracycline (30 μg), clindamycin (10 μg), vancomycin (30 μg), cefoxitin (30 μg), co-trimoxazole (1.25/23.75 μg), amikacin (10 μg), erythromycin (10 μg), cefepime (30 μg), levofloxacin (5 μg), and linezolid (30 μg). In contrast, for gram-negative bacteria, the antibiotics that were used included amikacin (30 μg), gentamicin (10 μg), ciprofloxacin (5 μg), cefotaxime(30 μg), imipenem (10 μg), cefoxitin (30 μg), ceftazidime (30 μg), piperacillin-tazobactam (100/10 μg), cefoperazone-sulbactam (75/30 μg), trimethoprim/sulfamethoxazole (1.225/23.75 μg), and fosfomycin (200 μg).

Phenotypic detection of beta-lactamase enzymes among gram-negative bacterial isolates

The isolates were screened for the phenotypic detection of enzymes such as ESBL, metallo-beta-lactamase (MBL), and AmpC beta-lactamase. For screening ESBL production, ceftazidime and ceftazidime plus clavulanic acid discs were used, with a difference of 5 mm in zone size between the two discs considered significant [10]. MBL production was detected using imipenem and imipenem-ethylenediaminetetraacetic acid (EDTA) discs, with a significant difference defined as a 7 mm inhibition zone [10]. For the detection of AmpC beta-lactamase, an inhibition zone difference greater than 4 mm between cefoxitin and cefoxitin plus cloxacillin discs was considered significant [11].

## Results

During the study period, all of the 180 samples of patients were received for culture and sensitivity. Among these patients, 74.45% were male and 25.55% were female. The samples included 48 CSF specimens, representing 26.67% of the total; 53 pleural fluid specimens, accounting for 29.45%; 23 peritoneal fluid specimens, making up 12.78%; 50 ascitic fluid specimens, representing 27.78%; four pericardial fluid specimens, constituting 2.22%; and one sample each of bile and synovial fluid specimens, each comprising 0.55% of the total. Of the 180 specimens, bacterial growth was observed in 32. The highest growth rate was in pleural fluid (n=11, 20.75%), while no growth was observed in bile and synovial fluid samples (Table 1).

**Table 1:** Analysis of bacterial growth in different kinds of body fluids CSF: cerebrospinal fluid

Body Fluids	Total No. (%)	Growth (%)	No Growth (%)
CSF	48 (26.67)	4 (8.33)	44 (91.67)
Pleural Fluid	53 (29.45)	11 (20.75)	42 (79.25)
Peritoneal Fluid	23 (12.78)	7 (30.43)	16 (69.57)
Ascitic Fluid	50 (27.78)	9 (18)	41 (82)
Pericardial Fluid	4 (2.22)	1 (25)	3 (75)
Bile	1 (0.55)	0 (0)	1 (100)
Synovial Fluid	1 (0.55)	0 (0)	1 (100)
Total	180	32 (17.77)	148 (82.23)

Out of 180 samples, 32 fluid specimens were culture-positive, with an isolation rate of 17.77%. Among the culture-positive, the frequency of gram-negative bacteria was 84% (27/32) and gram-positive bacteria was 16% (5/32) (Table 2).

**Table 2:** Distribution of gram-positive and gram-negative bacteria among body fluids

Organisms	Number		Percentage
Gram-positive	5		16%
Gram-negative	27		84%
Total	32	100%	

Of the 32 cultures-positive samples, *Escherichia coli* was the predominant isolated organism (n=9, 28.12%), followed by *Pseudomonas aeruginosa* (n=6,18.75%), *Klebsiella pneumoniae* (n=4, 12.5%), *Acinetobacter baumannii* (n=3, 9.37%), *Enterobacter cloacae* (n=2, 6.25%), coagulase-positive *Staphylococcus* (n=3, 9.37%). Less commonly isolated were *Streptococcus pneumoniae* (n=1, 3.13%) and *Enterococcus faecium* (n=1, 3.13%) (Table 3).

**Table 3:** Distribution of bacterial isolates in different body fluids CSF: cerebrospinal fluid

Bacterial isolates	CSF (%)	Pleural Fluid (%)	Peritoneal Fluid (%)	Ascitic Fluid (%)	Pericardial Fluid (%)	Total No. (%)
Escherichia coli	-	4 (44.44)	5 (55.56)	-	-	9 (28.12)
Pseudomonas aeruginosa	2 (33.3)	1 (16.6)	-	2 (33.3)	1 (16.8)	6 (18.75)
Klebsiella pneumoniae	-	-	2 (50)	2 (50)	-	4 (12.5)
Acinetobacter baumannii	-	2 (66.66)	-	1 (33.34)	-	3 (9.37)
Enterobacter cloacae	-	-	-	2 (100)	-	2 (6.26)
*Pseudomonas *species	-	2 (75)	-	1 (25)	-	3 (9.37)
Coagulase-positive* Staphylococcus*	1 (33.33)	2 (66.67)	-	-	-	3 (9.37)
Streptococcus pneumoniae	1 (100)	-	-	-	-	1 (3.13)
Enterococcus faecium	-	-	-	1 (100)	-	1 (3.13)
Total	4 (12.5)	11 (34.38)	7 (21.87)	9 (28.13)	1 (3.12)	32

Among gram-positive bacteria, coagulase-positive *Staphylococcus* was the most common bacterial isolate, with 100% sensitivity to vancomycin and maximum sensitivity to tetracycline (n=2, 66.67%). It showed 100% resistance to ciprofloxacin, cefoxitin, erythromycin, cefepime, levofloxacin, and ampicillin. *Streptococcus pneumoniae *showed 100% sensitivity to gentamicin, ciprofloxacin, and clindamycin but 100% resistance to tetracycline, cefoxitin, co-trimoxazole, erythromycin, and ampicillin. *Enterococcus faecium,* isolated from ascitic fluid, was sensitive to tetracycline, clindamycin, and co-trimoxazole and resistant to gentamicin, ciprofloxacin, cefoxitin, amikacin, and cefepime (Table 4).

**Table 4:** Antimicrobial susceptibility pattern of gram-positive bacteria S: sensitive; R: resistant

Antibiotic	Coagulase positive *Staphylococcus* (n=3)		*Streptococcus pneumoniae* (n=1)		*Enterococcus faecium* (n=1)	
	S (%)	R (%)	S (%)	R (%)	S (%)	R (%)
Gentamicin	1 (33.33)	2 (66.67)	1 (100)	0 (0)	0 (0)	1 (100)
Ciprofloxacin	0 (0)	3 (100)	1 (100)	0 (0)	0 (0)	1 (100)
Tetracycline	2 (66.67	1 (33.33)	0 (0)	1 (100)	1 (100)	0 (0)
Clindamycin	1 (33.33)	2 (66.67)	1 (100)	0 (0)	1 (100)	0 (0)
Vancomycin	3 (100)	0 (0)	1 (100)	0 (0)	1 (100)	0 (0)
Cefoxitin	0 (0)	3 (100)	0 (0)	1 (100)	0 (0)	1 (100)
Co-trimoxazole	1 (33.33)	2 (66.67)	0 (0)	1 (100)	1 (100)	0 (0)
Amikacin	1 (33.33)	2 (66.67)	1 (100)	0 (0)	0 (0)	1 (100)
Erythromycin	0 (0)	3 (100)	0 (0)	1 (100)	1 (100)	0 (0)
Cefepime	0 (0)	3 (100)	1 (100)	0 (0)	0 (0)	1 (100)
Levofloxacin	0 (0)	3 (100)	1 (100)	0 (0)	1 (100)	0 (0)
Linezolid	1 (33.33)	2 (66.67)	1 (100)	0 (0)	1 (100)	0 (0)
Ampicillin	0 (0)	3 (100)	0 (0)	1 (100)	1 (100)	0 (0)

Across all gram-negative bacterial isolates from sterile body fluids, *Escherichia coli *exhibited thehighest prevalence rate. *Escherichia coli* showed maximum sensitivity to gentamicin and fosfomycin (n=7, 77.78%) and maximum resistance to cefoperazone-sulbactam (n=8, 88.88%). *Pseudomonas aeruginosa* exhibited maximum sensitivity toward fosfomycin (n=5, 83.34%) and maximum resistance to gentamicin, cefotaxime, and cefoxitin, including other antibiotics (n=5, 83.34%). *Klebsiella pneumoniae* showed 50% sensitivity to amikacin, cefoxitin, ceftazidime, and other antibiotics, along with 100% resistance to cefotaxime. *Acinetobacter baumannii* showed 100% sensitivity to co-trimoxazole and fosfomycin and 100% resistance to gentamicin and cefotaxime.* Pseudomonas species *showed maximum sensitivity to amikacin, ceftazidime, fosfomycin, etc. (n=2, 66.66%) and showed maximum resistance toward gentamicin, ciprofloxacin, cefotaxime, etc. (n=2, 66.66%). *Enterobacter cloacae* showed 100% sensitivity to co-trimoxazole and fosfomycin and 100% resistance to imipenem, cefoxitin, and ceftazidime (Table 5).

**Table 5:** Antimicrobial susceptibility pattern of gram-negative bacteria S: sensitive; R: resistant

Antibiotics	*Escherichia coli* (n=9)		*Pseudomonas aeruginosa* (n=6)		*Klebsiella pneumoniae* (n=4)		*Acinetobacter baumannii* (n=3)		*Pseudomonas *species (n=3)		*Enterobacter cloacae* (n=2)	
	S (%)	R (%)	S (%)	R (%)	S (%)	R (%)	S (%)	R (%)	S (%)	R (%)	S (%)	R (%)
Amikacin	4 (44.44)	5 (55.56)	4 (66.67)	2 (33.33)	2 (50)	2 (50)	1 (33.34)	2 (66.66)	2 (66.66)	1 (33.34)	1 (50)	1 (50)
Gentamicin	7 (77.77)	2 (22.23)	1 (16.66)	5 (83.34)	1 (25)	3 (75)	0 (0)	3 (100)	1 (33.34)	2 (66.66)	1 (50)	1 (50)
Ciprofloxacin	3 (33.34)	6 (66.66)	2 (33.33)	4 (66.67))	1 (25)	3 (75)	1 (33.34)	2 (66.66)	1 (33.34)	2 (66.66)	1 (50)	1 (50)
Cefotaxime	2 (22.23)	7 (77.77)	1 (16.66)	5 (83.34)	0 (0)	4 (100)	0 (0)	3 (100)	1 (33.34)	2 (66.66)	1 (50)	1 (50)
Imipenem	2 (22.23)	7 (77.77)	2 (33.33)	4 (66.67))	1 (25)	3 (75)	2 (66.66)	1 (33.34)	1 (33.34)	2 (66.66)	0 (0)	2 (100)
Cefoxitin	3 (33.34)	6 (66.66)	1 (16.66)	5 (83.34)	2 (50)	2 (50)	2 (66.66)	1 (33.34)	0 (0)	3 (100)	0 (0)	2 (100)
Ceftazidime	0 (0)	9 (100)	1 (16.66)	5 (83.34)	2 (50)	2 (50)	1 (33.34)	2 (66.66)	2 (66.66)	1 (33.34)	0 (0)	2 (100)
Piperacillin-tazobactam	2 (22.23)	7 (77.77)	1 (16.66)	5 (83.34)	1 (25)	3 (75)	1 (33.34)	2 (66.66)	2 (66.66)	1 (33.34)	1 (50)	1 (50)
Cefoperazone-sulbactam	1 (11.12)	8 (88.88)	2 (33.33)	4 (66.67))	2 (50)	2 (50)	1 (33.34)	2 (66.66)	1 (33.34)	2 (66.66)	1 (50)	1 (50)
Co-trimoxazole	3 (33.34)	6 (66.66)	2 (33.33)	4 (66.67)	2 (50)	2 (50)	3 (100)	0 (0)	1 (33.34)	2 (66.66)	2 (100)	0 (0)
Fosfomycin	7 (77.77)	2 (22.23)	5 (83.34)	1 (16.66)	2 (50)	2 (50)	3 (100)	0 (0)	2 (66.66)	1 (33.34)	2 (100)	0 (0)

Among the 27 gram-negative bacterial isolates, *Escherichia coli *(n=5, 55.56%) were ESBL producers, followed by *Pseudomonas aeruginosa* (n=2, 33.33%), *Klebsiella pneumoniae* (n=1, 25%), *Enterobacter cloacae* (n=2, 100%), and *Pseudomonas species* (n=1, 33%). Notably, *Acinetobacter baumannii *was identified as a non-ESBL producer. Overall, the total number of ESBL producers in this study was (n=11, 40.74%) (Table 6).

**Table 6:** Prevalence of ESBL producing gram-negative bacteria ESBL: extended-spectrum beta-lactamase

Bacterial isolates	ESBL Positive (%)	ESBL Negative (%)
Escherichia coli	5 (55.56)	4 (44.44)
Pseudomonas aeruginosa	2 (33.33)	4 (66.67)
Klebsiella pneumoniae	1 (25)	3 (75)
Acinetobacter baumannii	0 (0)	3 (100)
Enterobacter cloacae	2 (100)	0 (0)
*Pseudomonas *species	1 (33)	2 (67)

Out of 27 gram-negative isolates,* Escherichia coli* (n=6, 66.67%), *Pseudomonas aeruginosa* (n=2, 33.33%), *Klebsiella pneumoniae* (n=2, 50%), and *Pseudomonas* species (n=1, 33%) were MBL producers. In contrast, *Enterobacter cloacae* was not identified as an MBL producer. The total number of MBL producers in this study was 13 (48.15%) (Table 7).

**Table 7:** Prevalence of MBL producing gram-negative bacteria MBL: metallo-beta-lactamase

Bacterial isolates	MBL Positive (%)	MBL Negative (%)
Escherichia coli	6 (66.67)	3 (33.33)
Pseudomonas aeruginosa	2 (33.33)	4 (66.67)
Klebsiella pneumoniae	2 (50)	2 (50)
Acinetobacter baumannii	2 (67)	1 (33)
Enterobacter cloacae	0 (0)	2 (100)
*Pseudomonas *species	1 (33)	2 (67)

Of the total 27 gram-negative bacterial isolates, *Pseudomonas aeruginosa *(n=2, 33.33%), *Klebsiella pneumoniae* (n=2, 50%), and *Pseudomonas species* (n=1, 33%) were identified as AmpC beta-lactamase (AmpC) producers. *Escherichia coli*, *Acinetobacter baumannii,* and *Enterobacter cloacae* were non-AmpC producers. The total number of AmpC producers in the current study was (n=5, 18.51%) (Table 8).

**Table 8:** Prevalence of AmpC producing gram-negative bacteria

Bacterial isolates	AmpC Positive (%)	AmpC Negative (%)
Escherichia coli	0 (0)	9 (100)
Pseudomonas aeruginosa	2 (33.33)	4 (66.67)
Klebsiella pneumoniae	2 (50)	2 (50)
Acinetobacter baumannii	0 (0)	3 (100)
Enterobacter cloacae	0 (0)	2 (100)
*Pseudomonas *species	1 (33)	2 (67)

Among the total 32 gram-positive and gram-negative bacterial isolates, 30 (93.75%) were identified as MDR. Out of all MDR organisms, *Escherichia coli* had the highest prevalence of 28.12%, followed by *Pseudomonas aeruginosa* with 18.75% and *Klebsiella pneumoniae *with12.5%. The least were *Enterobacter cloacae*, *Streptococcus pneumoniae,* and *Enterococcus faecium, *whichexhibited 3.13%, as shown in Table 9.

**Table 9:** Multidrug resistance pattern of bacterial isolates from sterile body fluids MDR: multidrug-resistant; RG0: resistant to zero groups of antibiotics, i.e., sensitive to all groups of antibiotics used; RG1: resistant to one group of antibiotics; RG2: resistant to two groups of antibiotics; RG3: resistant to three groups of antibiotics; RG4: resistant to four groups of antibiotics; >RG5: resistant to greater than or equal to five groups of antibiotics

Bacterial isolates	RG0	RG1	RG2	RG3	RG4	>RG5	MDR (RG3+RG4+>RG5) (%)
Escherichia coli	0	0	0	1	2	6	9 (28.12)
Pseudomonas aeruginosa	0	0	0	1	1	4	6 (18.75)
Klebsiella pneumoniae	0	0	0	1	1	2	4 (12.5)
Acinetobacter baumannii	0	0	1	0	2	0	2 (6.25)
Enterobacter cloacae	0	0	1	0	0	1	1 (3.13)
*Pseudomonas *species	0	0	0	1	0	2	3 (9.37)
Coagulase-positive *Staphylococcus*	0	0	0	0	0	3	3 (9.37)
Streptococcus pneumoniae	0	0	0	0	0	1	1 (3.13)
Enterococcus faecium	0	0	0	1	0	0	1 (3.13)
Total	0	0	2	5	6	19	30 (93.75)

**Figure 1:**
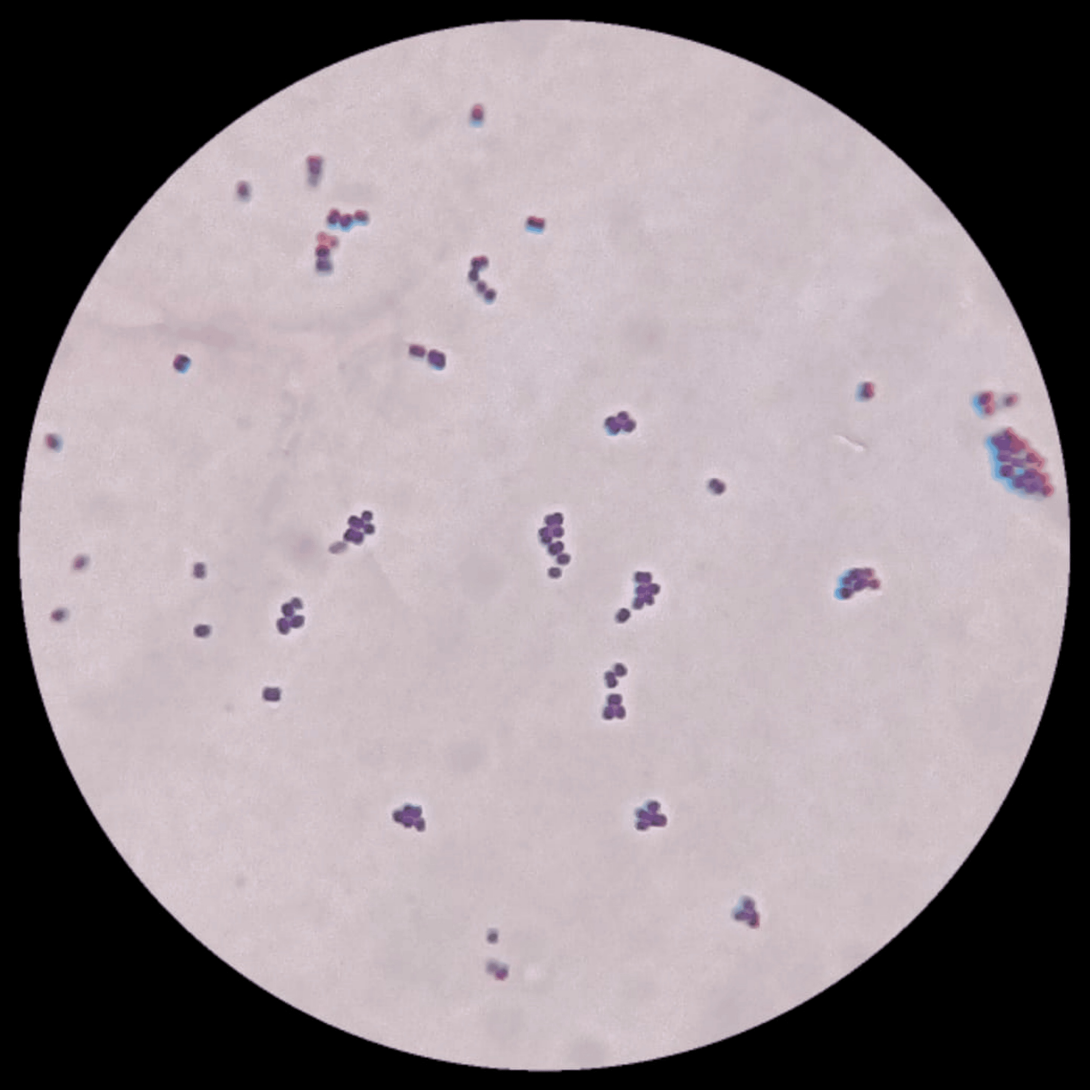
Gram-positive cocci (100X)

 Figure 1 shows the gram stain finding illustrating gram-positive cocci at 100X (oil immersion).

**Figure 2:**
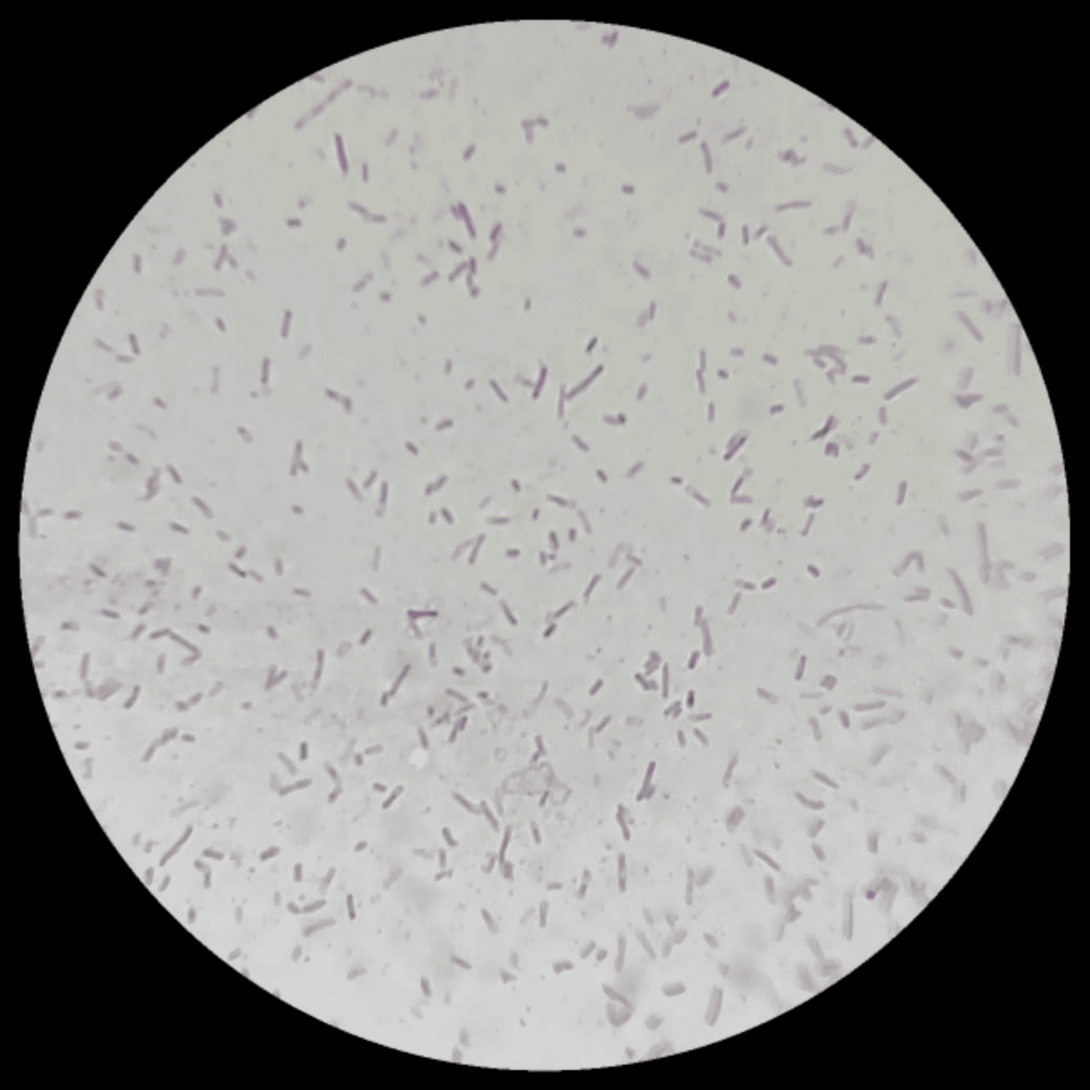
Gram-negative bacilli (100X)

 Figure 2 shows the gram stain finding demonstrating gram-negative bacilli at 100X (oil immersion).

## Discussion

At birth, humans are immediately exposed to a complex microbiome that plays a crucial role in their health. When humans are born, they acquire a diverse array of microorganisms, comprising bacteria, viruses, fungi, protozoa, and helminths [1]. This collective assembly of microorganisms associated with humans is commonly referred to as “microbiota” [12]. Nevertheless, certain tissues or organs of human body systems were traditionally believed to be sterile [1]. These sites and fluids typically do not harbor microorganisms as commensals when in a healthy state [2].

Systemic illness arises due to the invasion of different types of microorganisms in sterile sites of the body [13]. However, the isolation of microorganisms, including bacteria, viruses, fungi, and parasites (protozoa and metazoa), from these sites is considered harmful and can be life-threatening, particularly for critically ill patients in intensive care or high-dependency units of the hospitals [14].

In this study, the overall prevalence rate of both gram-positive and gram-negative bacterial isolates was 17.77%. This finding aligns with a previous study from India conducted in Telangana by Durga et al. wherein the prevalence rate of bacterial isolates was 20.55% [15]. The finding of the prevalence rate of bacterial isolates in the present study is highly similar to several previous studies carried out in Ethiopia, such as Eastern Ethiopia where Shume et al. reported 17% [13] and Northern Ethiopia where Tsegay et al. reported 20.2% [7]. As compared to the current study, a lower rate of prevalence was seen in several previous studies from Northwest Ethiopia by Admas et al. at 7.5% [16], in Balikesir, Turkey by Duran et al. at 9.7%[3], and in Uttar Pradesh, India by Singh et al. at 9.69% [17]. A higher prevalence rate of bacterial isolates as compared to the present study was found in Northern India in a study conducted by Kar et al. at 31.13% [18], in Nepal, Dharan, Sunsari by Shrestha et al. at 31% [5], and again in Bhubaneshwar, Odisha, India, the study conducted by Tiwari et al. reported 28% [14]. These variations may be due to differences in sample processing techniques, seasonal variation, and practice of infection control across distinct regions and studies.

According to the present study, the prevalence of gram-negative bacterial infection in sterile body fluids was predominantly observed at 84%, whereas infections caused by gram-positive bacteria constituted 16%. Similar findings were noted in the study by Rouf et al. wherein 70% were gram-negative bacteria and 30% were gram-positive bacteria [2], and the study by Durga et al. reported 67.81% gram-negative and 32.19% gram-positive bacteria [15]. In this study, the high prevalence rate of bacterial infection in sterile body fluids exhibited by gram-negative bacteria may be due to the durable outer membrane they possess, making them resistant to antimicrobial actions and immunological defenses. Their capacity to form biofilms and produce potent toxins further enhances their virulence.

Of the 32 culture-positive samples, *Escherichia coli* was the most frequently isolated organism demonstrating 28.12%, followed by* Pseudomonas* *aeruginosa* 18.75%, and in gram-positive bacteria coagulase-positive *Staphylococcus* 9.37%. Similar results were obtained by a couple of studies. As an example, in the study by Rouf et al., Shrestha et al., and Durga et al., *Escherichia coli* was the most common disease-causing bacteria in sterile body fluids according to the isolation rate of findings of all these studies [2,5,15].

In the present study, isolated organisms were tested against various antimicrobial agents and their susceptibility patterns were observed. Among gram-positive bacteria, coagulase-positive *Staphylococcus* was the dominant isolated organism and showed 100% susceptibility toward vancomycin and 66.67% susceptibility to tetracycline. These results are similar to Singh et al. who reported 100% sensitivity to vancomycin and 50% to tetracycline [17]. Equivalently Rouf et al. reported 100% sensitivity to vancomycin [2].

In the present study, coagulase-positive* Staphylococcus* showed 100% resistance toward ciprofloxacin, cefoxitin, erythromycin, cefepime, levofloxacin, and ampicillin. This finding aligns with the study conducted by Tsegay et al. who observed 78.6% resistance to ampicillin [7].

Among gram-negative bacterial isolates, *Escherichia coli* was in the majority and showed maximum susceptibility to both gentamicin and fosfomycin at 77.78%, followed by amikacin at 44.44%. These findings are similar to the study by Durga et al. wherein 85% susceptibility toward amikacin and 75% to gentamicin were seen [15]. Additionally, the study by Rouf et al. indicated that gentamicin and amikacin were the most effective antibiotics against *Escherichia coli *[2].

*Escherichia coli *showed maximum resistance to cefoperazone-sulbactam at 88.88%, followed by cefotaxime, imipenem, and piperacillin-tazobactam at 77.77%. These findings are similar to Sheikhbahaei et al. who found high resistance rates to both piperacillin-tazobactam and cefoperazone-sulbactam [19].

*Pseudomonas aeruginosa *was the second most etiological agent of sterile body fluid infections, exhibiting the highest sensitivity to fosfomycin at 83.34%, followed by amikacin at 66.67%. These findings are indistinguishable from those of Shume et al. and Durga et al. who reported 75% sensitivity to amikacin [13,15].

Among gram-negative bacterial isolates, 40.74% were ESBL producers. In a similar manner, Shrestha et al. reported 37% [5], and the study conducted by Singh et al. showed 25% of ESBL producers [17]. According to the present study, MBL was detected in 48.15% of cases, one out of two gram-negative bacterial isolates from CSF was either ESBL or MBL positive. The presence of ESBL and MBL isolates in the CSF in the present study indicates the development of resistant strains. Out of 27 gram-negative isolates, 18.51% were AmpC beta-lactamase positive. More prevalence of these resistant phenotypes in the present study might be attributed to increased use of beta-lactam antimicrobials in therapeutic management.

The rising prevalence of AmpC beta-lactamase, MBL, and ESBL-producing isolates signals a concerning trend of increasing resistance mechanisms among bacteria, which could render existing antimicrobial therapies less effective [20].

As per the present study, a massive and considerable prevalence rate of MDR organisms has been noticed. Of the total 32 bacterial isolates, (n=30, 93.75%) were multidrug-resistant. The present findings are enormously higher compared to those of Kar et al. where 45.13% of the isolated organisms from body fluids were multidrug-resistant [18]. Barely two isolates were not resistant to three or more than three antibiotic classes, other than these two isolates, the rest were MDR organisms. The matter of MDR organisms has been escalating notably due to overuse and misuse of antibiotics.

Limitations

Although the present study emphasized the bacteriological profile of sterile body fluids, infections may also involve anaerobic bacteria and non-bacterial microorganisms. However, anaerobic bacteria, eukaryotic microorganisms, and viruses were not detected in this study.

Additionally, the absence of molecular diagnostics, essential for confirming bacterial isolates and identifying genetic resistance mechanisms, was a limitation of this study. To improve the detection and characterization of microbial pathogens in sterile body fluids, it is recommended to incorporate advanced molecular techniques, such as a multiplex polymerase chain reaction (PCR) system, which enhances diagnostic capabilities by detecting different types of microorganisms from a single sample. Future research should prioritize molecular diagnostics to uncover genetic mechanisms of antimicrobial resistance.

## Conclusions

Infections of sterile body fluids caused by bacteria are critical due to their high mortality and morbidity rates. Instantaneous identification of the causative organisms and their antibiotic susceptibility is crucial for the survival of infected patients, particularly in cases of suspected meningitis. In this study, gram-negative organisms, specifically *Escherichia coli* and *Pseudomonas aeruginosa*, were the most common causes of sterile body fluid infections in the institution and showed sensitivity to gentamicin and fosfomycin. This suggests these drugs may be effective treatment options.

This study expresses the severity of MDR organism infection in sterile body fluids. Understanding the antimicrobial sensitivity pattern aids the judicious use of medications, thereby helping to prevent the development of drug resistance. Prompt initiation of appropriate antibiotic therapy can shorten hospital stays and minimize the emergence of drug resistance, making early diagnosis and treatment essential in clinical management strategies.
